# Intrinsic Functional and Structural Brain Connectivity in Humans Predicts Individual Social Comparison Orientation

**DOI:** 10.3389/fpsyt.2020.00809

**Published:** 2020-08-13

**Authors:** Wi Hoon Jung, Hackjin Kim

**Affiliations:** ^1^Department of Psychology, Daegu University, Gyeongsan, South Korea; ^2^Department of Psychology, Korea University, Seoul, South Korea

**Keywords:** diffusion tensor imaging, functional connectivity, resting-state fMRI, social comparison orientation, structural connectivity

## Abstract

Social comparison orientation (SCO), the tendency to compare oneself with others, is universal, varies widely across individuals, and predicts important life and health outcomes. However, the neural mechanism underlying individual differences in SCO is still not well-understood. In the present study, we identified intrinsic neural markers of SCO in healthy young adults (n = 42) using a multimodal neuroimaging approach that included diffusion tensor imaging and resting-state functional MRI data. We found that higher SCO was associated with weaker structural and functional connectivity (SC, FC) strengths between the ventral striatum and the medial prefrontal cortex, which are core regions of the brain reward network. Additionally, individual SCO was negatively associated with neural fluctuations in the intraparietal sulcus (IPS), part of the frontoparietal network, and positively with FC between the IPS and anterior insula/amygdala cluster. This finding was further confirmed by the observation of independently-defined, large-scale, inter-network FC between the frontoparietal network and cingulo-opercular network. Taken together, these results provide novel evidence for intrinsic functional and structural connectivity of the human brain associated with individual differences in SCO.

## Introduction

Social comparison—comparing one’s own opinions and abilities with those of others—is a constant and ubiquitous experience that occurs throughout life. People use social comparison to evaluate or groom their social reputation and relationships (1, 2). However, individuals vary in their tendency to engage in social comparison, and a person’s tendency to compare oneself with others is referred to as social comparison orientation (SCO). This can be measured using the Iowa-Netherlands Comparison Orientation Measure (INCOM) (3). Understanding individual differences in SCO is important, because higher SCO is associated with a variety of real-life behaviors and outcomes, including several psychological dimensions such as poorer self-perception and lower self-esteem (4), lower job satisfaction (5), more altruistic/helping behavior to unfamiliar others (6), and an increased susceptibility to mental illness such as depression (7). Despite its importance, the neural markers that may underlie individual differences in SCO have been only sparsely investigated.

Over the past decade, researchers have observed links between social comparison and reward processing at both the psychological and neurological levels. For example, social comparison is associated with increased activity in the ventral striatum (VS) and medial prefrontal cortex (MPFC) (8–12), both of which are considered core regions of the brain reward system (13–15). More recent studies have reported that functional communication between the VS and MPFC is critically involved in social comparison and may underlie individual differences in SCO (16, 17).

Besides the reward-related neural network, several other neural structures may be involved in general comparison in both social and non-social domains (12, 18). These regions include the intraparietal sulcus (IPS), anterior insula (aINS), and anterior cingulate cortex (ACC). The IPS is known to play a role in encoding numerical quantities and the activity of the region can be modulated by the distance between two magnitudes (i.e., comparison of numbers, size, luminance, or height) (19–21). The activity of the aINS and ACC increases when people compare themselves to better-off others, and it is stronger for self-other than for familiar-other comparisons (22, 23). However, it is not yet clear whether structural and functional features of these areas at rest are linked to individual variability in SCO.

Recently, several studies have used task-independent neural measures to predict specific behaviors. These measures are more likely to be of practical use, because they are likely less tied to a specific context and are therefore relatively stable over time (24, 25). However, to our knowledge, no neuroimaging studies have yet investigated task-independent neural markers of individual differences in SCO. In the present study, we thus examined neural predictors of individual differences in SCO using a multimodal, task-independent neuroimaging approach, including diffusion tensor imaging (DTI), and resting-state functional magnetic resonance imaging (RS-fMRI), that focused on individual variations in the brain’s intrinsic structural and functional network architecture. Because the reward network plays a central role in social comparison, we first aimed to identify potential associations between individuals’ SCO scores and the strength of structural connectivity (SC) and resting-state functional connectivity (FC) between the VS and the MPFC, which are the central components of the reward network (i.e., intra-network connectivity within the reward network). For exploratory purposes to test whether SCO is associated with other areas and networks outside the reward network, we next performed the following additional analyses at the whole-brain voxel level using data-driven approach examining RS-fMRI and DTI data. In other words, to identify neural signatures of SCO at the whole-brain voxel level, we searched for brain areas associated with individual SCO in terms of local features (e.g., local connectivity and local fluctuation) in neural activity and in SC. This analysis highlighted the IPS, the neural structure previously linked to aspects of general comparison. To further characterize this association, we searched for neural regions whose FC strength with the IPS is associated with SCO, by using seed-based FC analysis, and found a significant association between the IPS–aINS/amygdala FC strength and SCO. Given that core areas of the frontoparietal network (FPN) and of the cingulo-opercular network (CON) include the IPS and aINS (26, 27) respectively, we investigated whether individual SCO scores are associated with the inter-network FC between these two large-scale functional networks. Finally, linear regression analyses with identified neural variables showed that each of the identified features, particularly that from the RS-fMRI data, uniquely explains the variance in SCO. These results provide a set of unique multi-modal intrinsic neural markers associated with individual differences in SCO.

## Materials and Methods

### Participants

A total of 47 participants were recruited from Korea University and the surrounding community. From each participant, we collected high-resolution T1-weighted anatomical MRI, RS-fMRI, DTI, and fMRI during an incentive delay task in the context of social comparison. In this study, we focused on task-independent measures of brain function and structure (i.e., RS-fMRI and DTI data) to examine the link between individual variations in SCO and individual differences in intrinsic functional and structural brain features. Among all, 43 had both DTI and RS-fMRI data available, and four participants were lost due to technical problems. One additional participant was excluded due to excessive head motion during RS-fMRI—that is, > 2.5 mm of translation or 2.5° of rotation and > 0.24 mm mean frame-wise displacement (FD; > 2 standard deviations from the group mean) (28). Ultimately, the data of 42 participants [27 women, 15 men, age (mean ± SD): 22.29 ± 3.04 years, all right-handed, SCO: 3.79 ± 0.58] were used in the final analyses. We confirmed that the final sample size was rational to obtain scientifically meaningful results, based on a power analysis performed before data analysis using G*Power software (29). Assuming an effect size of 0.5, an alpha level of 0.05, and a power of 0.90 to ensure correlation with the bivariate normal model, the G*Power analysis resulted in a required sample size of 37. All study procedures were approved by the Institutional Review Board of Korea University, and all participants provided written informed consent.

### Measuring Social Comparison Orientation

The degree of SCO for each participant was assessed using the Iowa-Netherlands Comparison Orientation Measure scale (INCOM) (2, 3), which is a widely used scale to test an individual’s SCO. It consists of 11 items, each scored using a 5-point Likert scale (1 = I disagree strongly, 5 = I agree strongly). The INCOM measures an individual’s tendency toward social comparison (e.g., “I often compare myself with others with respect to what I have accomplished in life”). All participants filled out the debriefing questionnaires, including the INCOM, scale before completing the scans.

### Image Acquisition

All images were scanned using a 3-T scanner (Siemens Magnetom Trio; Erlangen, Germany). High-resolution, T1-weighted anatomical images were acquired using a 3D magnetization-prepared, rapid-acquisition gradient echo (MPRAGE) sequence [repetition time (TR) = 1,900 ms, echo time (TE) = 2.52 ms, flip angle (FA) = 9°, voxel size = 1.0 × 1.0 × 1.0 mm, 192 sagittal slices]. Next, functional images were obtained using T2*-weighted, echo-planar imaging (EPI; TR = 2,000 ms, TE = 20 ms, FA = 90°, voxel size = 3.0 × 3.0 × 3.0 mm, 42 interleaved axial slices, and 155 volumes). During RS-fMRI, participants were instructed to keep their eyes open and maintain fixation. An eye-tracker mounted on a head coil was used to monitor the participants’ eyes and ensure they did not fall asleep during the scan. Finally, DTI data were acquired with a 32-channel head coil using a single-shot, multiband EPI sequence (TR = 3,000 ms, TE = 70 ms, FA = 90°, multiband acceleration factor = 3, phase partial Fourier = 6/8, voxel size = 2.0 × 2.0 × 2.0 mm, 75 interleaved axial slices, and 64 diffusion directions with b-values of 1,000 s/mm^2^ and 8 images with b-values of 0 s/mm^2^).

### Structural Connectivity Analysis Within the Reward Network

**Figure 1:**
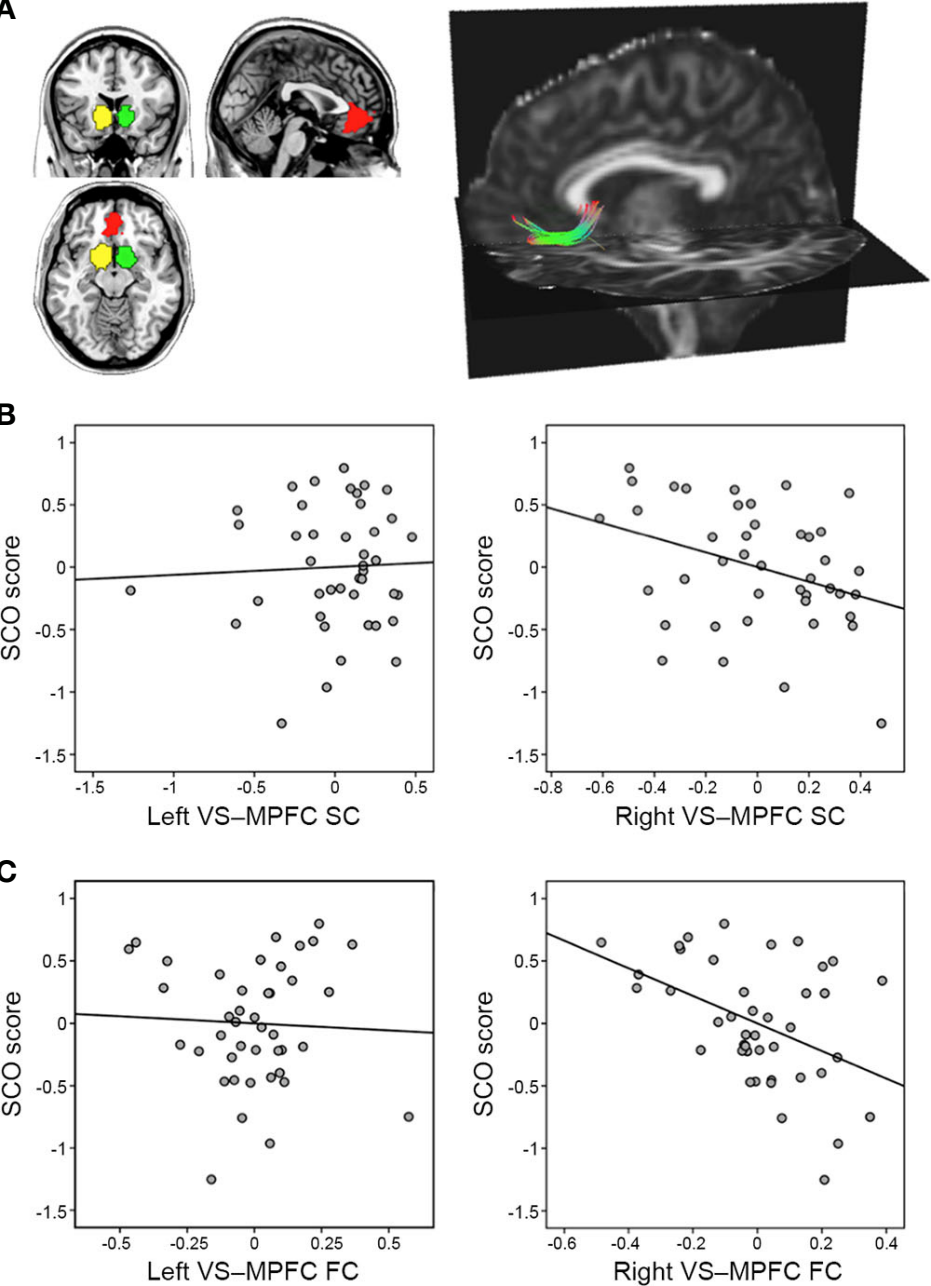
Associations between social comparison orientation (SCO) and the reward network. **(A)** The right panel shows three regions of interest—the left ventral striatum (VS; yellow), the right VS (green), and the medial prefrontal cortex (MPFC; red). The left panel displays a single subject’s tracking map between the right VS and the MPFC, identified using tractography analysis (for illustration purposes only). **(B)** Partial correlation scatterplot between SCO score and normalized fiber number, displayed as the strength of structural connectivity between the VS and MPFC. **(C)** Partial correlation scatterplot between SCO score and VS–MPFC functional connectivity strength (z-transformed). For illustration purposes, **(B, C)** were generated using Pearson’s correlation analysis between residuals after age and sex were regressed out.

DTI data were preprocessed using PANDA v1.3.1 (30) (https://www.nitrc.org/projects/panda/): a pipeline tool for diffusion MRI that uses the processing functions of established packages, including FSL (https://fsl.fmrib.ox.ac.uk/fsl/fslwiki/) and the Diffusion Toolkit (https://www.nitrc.org/projects/trackvis/). Briefly, a brain mask was made using the b0 images. Diffusion images were registered to the average of the b0 images using an affine transformation to correct for eddy current-induced distortions and simple head-motion. Whole-brain fiber tracking was performed using the fiber assignment by continuous tracking (FACT) algorithm (31), with the fractional anisotropy threshold set at 0.20 and the tracking turning angular threshold set at 45°. Afterwards, spline filtering was applied to smooth the streamline tractography. To quantify the degree of connection between the left VS and MPFC, as well as between the right VS and MPFC in the native space, we first identified these three regions-of-interest (ROIs) based on a previous meta-analysis involving the valuation system in the human brain (**Figure 1A**) (32). Next, these ROIs were transformed from the Montreal Neurological Institute (MNI) space to each subject’s native space. The number and average length of the fibers connecting each pair of ROIs were then calculated (**Figure 1B**). To normalize the fiber number, we divided it by the average volume and length of the two connecting regions. This counteracted bias where it was larger; closer brain regions inherently project/receive more fibers. Because the values were non-normally distributed, they were log-transformed before subsequent statistical analysis. We performed partial correlation, with age and sex as covariates, between SCO scores and normalized fiber numbers.

### Functional Connectivity Analysis Within the Reward Network

In the case of RS-fMRI data, the first five volumes were discarded to avoid instability in the initial data signal. Preprocessing steps, which included slice-acquisition timing, motion correction, nuisance signal regression, and spatial normalization, were performed using SPM12 (www.fil.ion.ucl.ac.uk/spm) and DPARSFA toolbox (33) (www.rfmri.org/DPARSF). To remove the effects of head motion and non-neuronal fluctuations on signals, as in recent studies (34), the following nuisance parameters were included as regressors within the general linear model: Friston 24-motion parameters, five principal components estimated from both the white matter and cerebrospinal fluid regions using a component-based noise correction method (35), mean global signal, head motion scrubbing regressors one volume before and two volumes after the bad time point (root mean square volume-to-volume displacement > 0.25) (36), and two polynomial trends (linear trend and quadratic trend). The spatially normalized residual images were smoothed using a 6-mm Gaussian kernel and band-pass filtering (0.01–0.1 Hz). The Pearson correlation coefficients were then computed between the mean time series of an ROI pair (i.e., MPFC, left VS, and right VS) as the strength of FC. The correlations were then converted to z-values using Fisher r-to-z transformation. We also performed partial correlation (covariates: age, sex, and motion indexed by mean FD) between SCO scores and z-transformed FC values, with the following co-variates.

### Exploratory Voxel-Level Whole-Brain Analysis

To ensure the study was complete and identify the brain areas or networks, mentioned in the *Introduction*, that are associated with individual SCO, we performed multiple regression analysis on data-driven structural and functional brain maps generated from DTI and RS-fMRI data together with SCO scores. Specifically, for voxel-level whole-brain DTI analysis, preprocessed DTI images were used to estimate four DTI metrics in the DTIFIT function of FSL: fractional anisotropy (FA), which measures the directionality of water diffusion, axial diffusivity (AD), which measures diffusion parallel to the white matter tract, radial diffusivity (RD), which measures diffusion perpendicular to the tract, and mean diffusivity (MD), which measures the diffusion speed of water molecules. The MD was estimated as the mean of all three eigenvalues [(λ1+ λ2+ λ3)/3], RD as the mean of the second and third eigenvalues [(λ2+ λ3)/2], and AD as the principal eigenvalue (λ1). To estimate the voxel-wise values of the DTI metrics of each subject’s skeleton, we performed tract-based spatial statistics (TBSS) (37). All the subjects’ FA images were aligned into the MNI standard space using the non-linear registration tool FNIRT. Next, a mean FA image was created and skeletonized/thinned to produce an image representing the center of all tracts common to the group (threshold = 0.2) (**Figure S1**). Each subject’s aligned data (including FA, AD, RD, and MD) were then projected onto this skeleton. Finally, for the FA, AD, RD, and MD maps, we performed permutation-based statistics (using FSL’s randomize with 5,000 permutations) to determine the areas in which DTI metrics were associated with SCO. Age and sex were included as covariates. Threshold-free cluster enhancement (TFCE) was used to correct for multiple comparisons (corrected *p* < 0.05).

For voxel-wise, whole-brain RS-fMRI analysis, the following local FC maps were generated with a default setting of DPARSFA: i) regional homogeneity (ReHo)—a measure of localized intraregional connectivity (38), ii) DC—a measure of local network connectivity (39, 40), and iii) fractional amplitude of low-frequency fluctuations (fALFF)—a measure of the relative contribution of specific local frequency fluctuations in neural activity to the whole frequency range (41). The ReHo maps were created using the Kendall coefficient of concordance of each voxel’s time series with those of its 26 neighboring voxels (38). The DC maps were obtained by summing of the weights of the significant connections (*r* > 0.25) (39, 40) for each voxel. For each of these three RS-fMRI maps, we implemented multiple regression analysis in SPM to identify areas in which the values of each map were associated with SCO, controlling for age, sex, and mean FD as covariates.

Using the IPS cluster that had significant fALFF activity in the whole-brain RS-fMRI analysis, we generated and analyzed seed-based FC maps that were seeded using IPS. The maps were regressed against SCO scores to identify regions that were functionally coupled with IPS as a function of individual SCO differences. All results were corrected for multiple comparisons to a significance level of *p* < 0.05 [uncorrected height threshold of *p* < 0.001 combined with a family-wise error (FWE)-corrected extent threshold of *p* < 0.05].

### Mediation Analysis With the Neural Features From the Exploratory Whole-Brain Analyses

To further examine the relationship between SCO and the above IPS findings (i.e., IPS fALFF and IPS–aINS/amygdala seed-based FC), we tested whether the direct effect of the IPS fALFF strength (X) on SCO (Y) could be explained in terms of the indirect influence of IPS–aINS/amygdala FC strength (M) as a mediator. To this end, we used the M3 Mediation Toolbox (https://github.com/canlab/MediationToolbox). Age, sex, and mean FD were included as covariates. Bootstrapping with 10,000 resamples was used for statistical inference in each path (*p* < 0.05).

### Inter-Network Connectivity Between the Frontoparietal and Cingulo-Opercular Networks

Based on the results of the multiple regression analysis with the above seed-based FC maps, we hypothesized that SCO is associated with functional interactions between two largely independent neural networks: the FPN and the CON, also often referred to as the salience network, because the IPS and aINS/amygdala clusters reported above are the core regions of these two networks, respectively (26, 27). To validate this hypothesis, we evaluated the data within a network framework. Specifically, the nodes of each network (25 nodes in the FPN and 14 nodes in the CON) consisted of 6-mm radius spheres centered on the coordinates taken from the corresponding networks in the Power-264 atlas, as defined in terms of the task-based fMRI and resting-state FC techniques (28). Next, to estimate inter-network FC, we extracted the mean time series from each of the nodes, computed the average connectivity across all node-to-node connections between the two networks using Pearson’s correlation, and converted the correlations into z-values using Fisher r-to-z transformation. For exploratory purposes, we also computed the average connectivity across node pairs within the same network, defining this as intra-network FC. We then performed partial correlation (covariates: age, sex, and mean FD) between SCO scores and z-transformed FC strengths.

### Testing the Effectiveness of Neural Predictors of Social Comparison Orientation

Finally, we examined whether the neural features found in this study (i.e., right VS–MPFC SC, right VS–MPFC FC, fALFF in the IPS, IPS–aINS/amygdala FC, and inter-network FC between FPN and CON) explain independent or overlapping variance in SCO. This was done by performing a multiple linear regression analysis including all of the neural features as independent variables to explain the variance in SCO. Notably, significant features in the multiple regression model explain variance in SCO over and above that explained by all other remaining features (25). We used SPSS Statistics version 25 to perform the linear regression analysis on each brain variable alone, as well as on all five identified brain variables together. Before performing this statistical analysis, the effects of age and sex were regressed out of all the neural variables.

## Results

### Intrinsic Structural and Functional Connectivity Within the Reward Network

The SCO scores were negatively associated with both SC (*r* = −0.350, *p* = 0.027) and FC (*r* = −0.479, *p* = 0.001) between the right VS and the MPFC, whereas no correlation was found in the left hemisphere (SC: *r* = 0.044, *p* = 0.787; FC: *r* = 0.054, *p* = 0.734) (**Figure 1**). There were no significant correlations between the strengths of SC and FC (*r* = −0.134, *p* = 0.399 for the left VS–MPFC connection; *r* = 0.133, *p* = 0.403 for the right).

### Exploratory Voxel-Wise Whole-Brain Analysis

**Figure 2:**
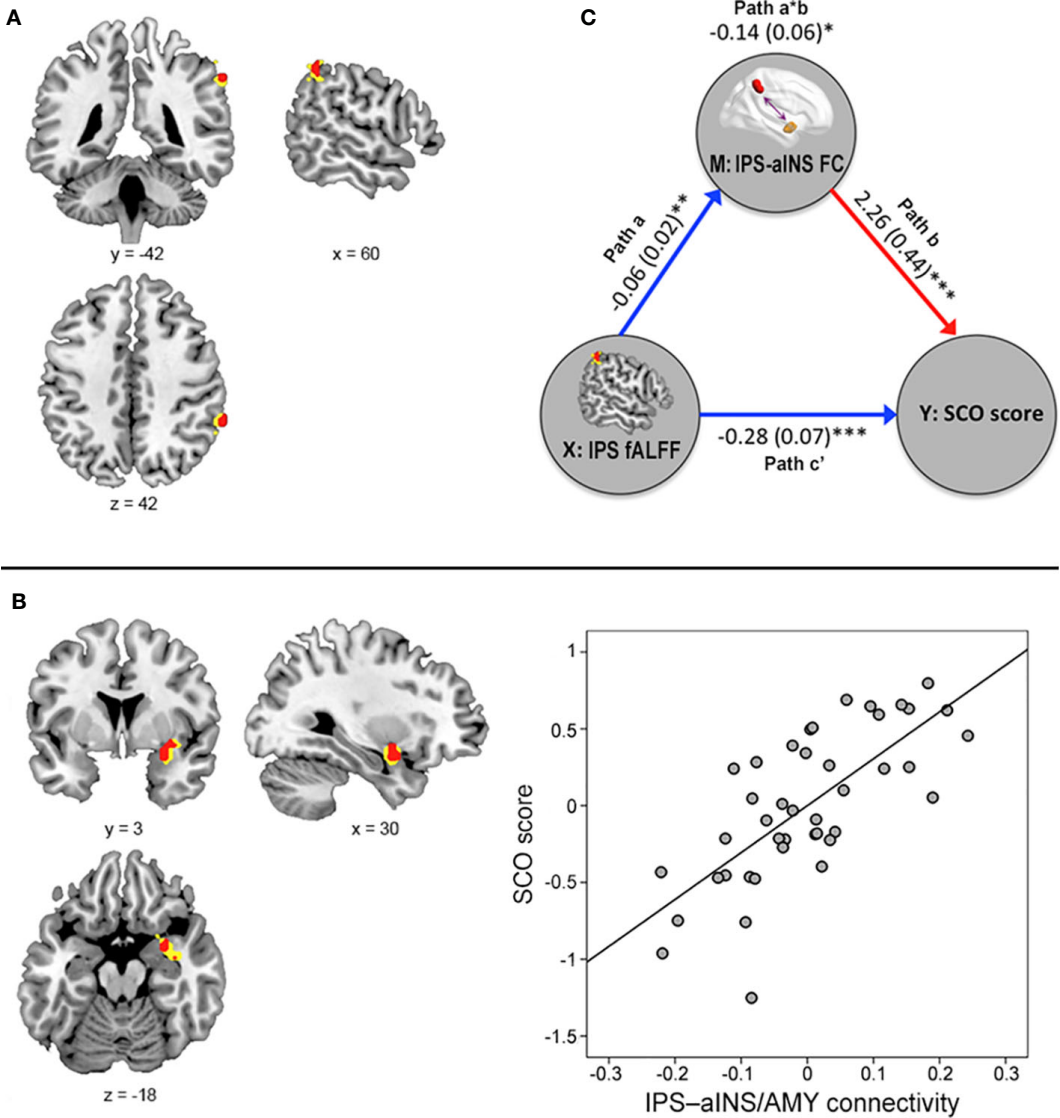
Results from the voxel-level, whole-brain resting-state functional magnetic resonance imaging analyses. **(A)** The fractional amplitude of low-frequency fluctuation (fALFF) value in the right intraparietal sulcus (IPS) was negatively associated with social comparison orientation (SCO) score [height *p* < 0.001 (red) or *p* < 0.005 (yellow)]. **(B)** Functional connectivity strength between the right IPS seed and right anterior insula (aINS)/amygdala cluster was positively associated with SCO score [height *p* < 0.001 (red) or 0.005 (yellow)]. For illustration purposes, this scatterplot was generated by performing Pearson correlation analysis between residuals age, sex, and motion were regressed out. **(C)** The mediation effect of functional connectivity strength in the IPS–aINS/amygdala on the right IPS fALFF and SCO scores. All paths (paths a, b, and c’) and mediation effects (path a*b) are labeled with path coefficients and their standard errors in parenthesis. Blue and red arrows indicate negative and positive relationships, respectively. *p < 0.05, **p < 0.01, ***p < 0.001.

Voxel-level whole-brain RS-fMRI analysis revealed that the fALFF value in the right IPS (peak MNI x, y, z coordinates = 60, −42, 42; peak z-value = 4.45) was negatively associated with SCO score (**Figure 2A**). No regions showed any significant correlation with other voxel-level whole-brain RS-fMRI maps including ReHo and DC maps at an uncorrected significance level of *p* < 0.001 and a FWE-corrected extent of *p* < 0.05.

Further multiple regression analysis using seed-based FC maps, with the right IPS acting as the seed point, revealed that SCO score was positively associated with FC strength between the right IPS seed and right aINS extending to amygdala (referred to as “aINS/amygdala” cluster here), areas belonging to the FPN and CON respectively (x, y, z coordinates = 30, 3, −18; z-value = 3.95; uncorrected significance level *p* < 0.001; FWE-corrected extent *p* < 0.05; **Figure 2B**).

No regions showed any significant correlation with the whole-brain structural maps created using DTI data (FA, MD, AD, and RD TBSS maps).

### Mediation Effect

**Figure 2C** shows the mediation effect of IPS–aINS/amygdala FC on the relationship between SCO score and fALFF in the IPS. In particular, fALFF was negatively correlated with the FC between IPS and aINS/amygdala (path a). The same FC was positively correlated with SCO score (path b). Finally, IPS–aINS/amygdala FC exhibited a negative mediation effect (negative path a*b) that resulted from an IPS fALFF-associated reduction in IPS–aINS/amygdala FC (negative path a), and there was a positive relationship between IPS–aINS/amygdala FC and SCO score (positive path b). This finding indicates that stronger FC between the IPS and the aINS/amygdala mediates the reduced fALFF in the IPS among individuals with high SCO scores.

### Social Comparison Orientation Associated With Inter-Network Connectivity

**Figure 3:**
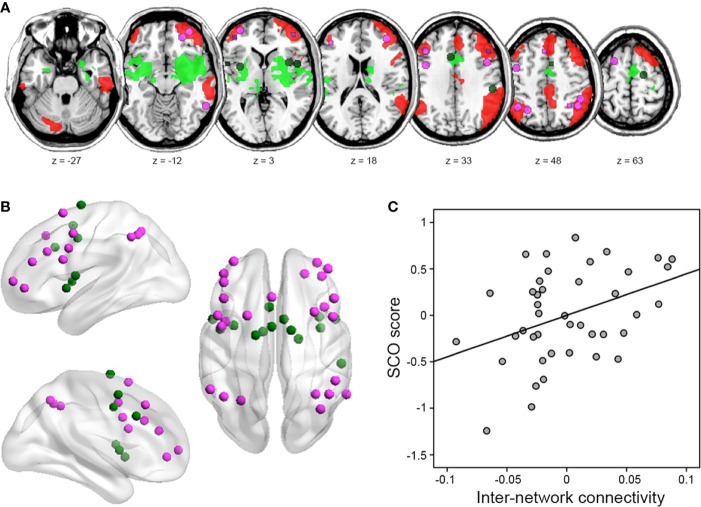
Results of inter-network connectivity analysis. **(A)** Figure illustrating the location of regions (i.e., nodes) in the frontoparietal network (FPN) (violet) and cingulo-opercular network (CON) (green), identified from the atlas by Power *et al*. (42). **(B)** Identified nodes overlaid on within-group seed-based functional connectivity map of each right IPS and right aINS/amygdala seed, identified from voxel-level whole-brain analysis (uncorrected height *p* < 0.001 and FWE-corrected extent *p* < 0.05). **(C)** Partial correlation between SCO score and FPN–CON inter-network connectivity strength. For illustration purposes, this scatterplot was generated using Pearson correlation analysis between residuals after age, sex, and motion were regressed out.

In line with our hypothesis, SCO score correlated positively with inter-network connectivity strength between the FPN and CON (*r* = 0.393, *p* = 0.013; **Figure 3**). An exploratory analysis with intra-network connectivity revealed that there were no associations between SCO score and intra-network connectivity (*r* = 0.282, *p* = 0.082 for the FPN; *r* = 0.223, *p* = 0.173 for the CON).

### Regression Models Predicting Individual Difference in Social Comparison

A linear regression model using all neural variables revealed that the FC within the reward network (right VS–MPFC FC), neural fluctuation (i.e., fALFF) in IPS activity, and the FC between the IPS and aINS/amygdala were significant predictors (*p* < 0.05) of SCO (**Table 1**). In such a combined model, significant measures explain the variance in SCO more than all other measures. We also ran linear regressions with each of the measures individually, allowing us to compare the variance explained by each measure (**Table 1**). The variance (*R^2^*) estimated from these analyses, arranged in ascending order, was as follows: 0.12 for the right VS–MPFC SC alone, 0.14 for the FPN–CON inter-network FC alone, 0.19 for the right VS–MPFC FC alone, 0.45 for the fALFF in the IPS alone, and 0.52 for the IPS–aINS/amygdala FC alone. The variance of the combined model was 0.75.

**Table 1:** Summary of linear regression models with each brain measure individually^1^ and together.

	Model withVS–MPFC SC	Model withVS–MPFC FC	Model withfALFF in IPS	Model withIPS–aINS/AMY FC	Model withFPN–CON FC	Model with allbrain variables
Constant	−2.38E−5 (0.07)	−2.46E−5 (0.07)	−2.38E−5 (0.06)	−2.24E−5 (0.05)	−2.18E−5 (0.07)	−2.25E−5 (0.04)
VS–MPFC SC	−0.59 (0.25)*	–	–	–	–	−0.12 (0.15)
VS–MPFC FC	–	−1.10 (0.36)**	–	–	–	−0.51 (0.22)*
fALFF in IPS	–	–	−0.43 (0.08)***	–	–	−0.26 (0.06)***
IPS–aINS/AMY FC	–	–	–	3.05 (0.47)***	–	1.83 (0.42)***
FPN–CON FC	–	–	–	–	4.18 (1.64)*	1.56 (1.02)
*R^2^*	0.12	0.19	0.45	0.52	0.14	0.75

^1^Linear regressions with each individual measure were performed for comparison purposes, with the amount of the variance (R^2^) explained in terms of the model with all brain variables.

Data are given as unstandardized coefficients, B (standard errors).

Coefficients significantly different from zero are indicated by asterisks: *p < 0.05, **p < 0.01, ***p < 0.001.

R^2^ values indicate the amount of variance, explained by the model.

VS–MPFC SC, structural connectivity strength between right ventral striatum and medial prefrontal cortex, as part of the reward network; VS–MPFC FC, functional connectivity strength between right ventral striatum and medial prefrontal cortex; fALFF in IPS, fractional amplitude of low-frequency fluctuation in intraparietal sulcus; IPS–aINS/AMY FC, functional connectivity strength between IPS and anterior insula/amygdala; FPN–CON FC, inter-network functional connectivity between the frontoparietal network and cingulo-opercular network.

## Discussion

In the present study, we investigated whether individual differences in social comparison, as measured by SCO, were related to multimodal, context-independent, brain measures, estimated using DTI and RS-fMRI data. In so doing, we identified several intrinsic functional and structural neural markers of SCO. Most importantly, individuals with higher SCO showed weaker SC and FC between the right VS and the MPFC—regions belonging to the reward-related neural network. We also found several exploratory results from the whole-brain voxel level analyses and network analysis. Individuals with higher SCO showed reduced spontaneous neural activity in the IPS—a region belonging to the FPN, increased FC between the IPS and aINS/amygdala, and large-scale inter-network FC between the FPN and CON. Of these measures, right VS–MPFC FC, fALFF in IPS, and IPS–aINS/amygdala FC contributed most to the neural prediction of SCO. The predictive model using all neural markers identified in the present study was highly effective—accounting for a substantial amount of variance in SCO (*R^2^* = 0.75). Taken together, these findings suggest that individual differences in social comparison can be characterized in terms of specific patterns in neural structures as well as intrinsic neural activity, particularly in the neural networks engaged in reward processing and comparative processing of external stimuli.

Our findings of SC and FC between the right VS and MPFC are broadly consistent with previous studies linking the same markers with reward processing. Both the VS and MPFC play a critical role in reward processing, showing elevated activity in response to both primary (e.g., food) and secondary reward stimuli (e.g., money) (43, 44). Importantly, these two regions contribute to the appraisal or representation of the subjective value of either social or non-social rewards (32, 42, 45, 46), providing strong evidence for a common neural currency (47, 48). Relatedly, activity in the VS and MPFC are modulated by the absolute outcome and by the relative payoff differences derived from social comparison (8, 9, 49), and this mechanism can vary depending on cultural membership (17). Several studies have indicated that functional interaction between the VS and MPFC reflects variability in the behavioral changes caused by social comparison (16, 17). For example, in one study, the VS response to social gains (winning more than a counterpart) during the earlier outcome phase predicted MPFC activity during the subsequent decision phase, and experienced social gains induced behavioral changes in later trials (16). In addition, the VS-MPFC FC strength predicted individual variability in the degree to which participants’ decisions were affected by relative income (17).

In this study, the association of SCO with SC and FC between VS and MPFC was significant only in right hemisphere. Though this was not expected, many previous studies have proposed hemispheric specialization of the reward network and of social processing. For instance, a recent functional MRI meta-analysis study shows that hemispheric dominance of striatum activation varies across different types of reward, including food, erotic, and money stimuli (50). In addition, a right-lateralized connectivity of the VS to the parietal cortices during resting-state has been reported (51). Studies in different types of social contexts demonstrate right hemisphere superiority in processing and detecting social stimuli (e.g., voices, faces, and gestures) (52, 53) as well as understanding the intentions behind other’s actions (54, 55). Future neuroimaging studies combining both behavioral task on social comparison and resting-state fMRI with a larger sample size will help to verify the observed hemispheric lateralization of FC associated with social comparison.

The present study showed that higher social comparison was associated with weaker VS-MPFC FC during resting-state. Previous studies have reported the involvement of VS-MPFC FC in the manifestation of clinical symptoms, such as addiction and depression (56, 57), as well as reward learning and valuation (16, 58). In the studies on social comparison, an increased activity and FC within the reward network have been observed specifically when people compare themselves to worse-off others (referred to as downward comparison), which is often associated with positive feelings (17, 23). Thus, it can be speculated that such a hypo-connectivity in the reward network during resting-state observed in the present study may reflect reduced baseline intrinsic reward sensitivity, which may cause people to seek excessive extrinsic social rewards possibly through increased social comparison to others, leading to positive (downward comparison) as well as negative (upward comparison) feelings. In line with this explanation, patients with hyperactivity or increased reward-seeking behavior showed reduced neural responsiveness in the VS, a key part of the reward network (59). Notably, usage-dependent selective synapse elimination (60) is often observed as an example of day-to-day experience-dependent neural plasticity (61), which may be the mechanism underlying decreases in neural activity and cortical thickness after training (17). Another possible explanation is that the reduced SC and FC between the VS and MPFC may indicate that the number of available alternatives is reduced because subjects engage in the excessive pursuit of a limited number of rewards. One good example of such a state may be approval addiction, which involves the excessive pursuit of approval to gain superior social status to others (i.e., downward comparison). The desire of social approval may be the main cause of social comparison. Though we speculate above on interpretations for our findings, we caution against these interpretations as we did not have any behavioral data to prove these interpretations. Therefore, further study may be necessary to investigate whether weaker resting-state VS–MPFC FC is associated with FC in the same circuit during certain social comparison behavior. Such research would provide a more accurate understanding of the functional implication of VS–MPFC FC in social comparison.

In the present study, individuals with higher SCO exhibited less fALFF in the right IPS, which is part of the FPN. While FC quantifies temporal synchrony between remote brain areas, the fALFF indicates quantifiable magnitudes of spontaneous regional neural activity across the whole brain (41). In other words, the fALFF allow us to probe local brain regions where individual differences in resting state activity are correlated with their phenotype (in this case, SCO) across the whole brain at voxel level. The IPS plays a crucial role in visuospatial attention and arithmetic processing (62), and it activates during cognitive and perceptual comparison of stimuli that differ in various ways (e.g., number, size, or luminance) (19–21). Notably, previous studies have demonstrated that the degree of IPS activity increases with the difficulty of comparison (20, 63). IPS activity is also increased during the comparison of social status (63), as well as during comparison of one’s own height against those of acquaintances (26). Interestingly, using the IPS as the seed region, further regression analysis between the IPS seed-based FC maps and the SCO revealed that higher SCO was associated with greater FC between the IPS seed and the aINS/amygdala cluster. Additionally, IPS–aINS/amygdala FC partially mediated the link between fALFF in the IPS and the SCO. Therefore, our findings suggest that the SCO is associated with various features of intrinsic neural activity in the IPS, including the power of local neural activity and the patterns of FC.

Considering that the IPS and aINS/amygdala are the core regions of the FPN and CON, respectively, we examined inter-network FC between the FPN and CON, which were independently identified in a previous study (28). Thus, we confirmed that higher SCO scores are associated with stronger inter-network FC between the FPN and CON. The aINS/amygdala cluster, which comprises key elements of the CON, together with the ACC, has been strongly implicated in social and non-social emotions, including disgust (64), pain (65), unfairness (66), and empathy (67), and interoceptive and emotional awareness (68–70), as well as in saliency detection (71). Relevant to the present study, the aINS is often engaged during social comparison (22, 23). In particular, a recent meta-analysis of functional neuroimaging studies emphasized the roles of the aINS and ACC in upward comparison (23). Previous studies have reported competitive and cooperative interactions between FPN and CON (72, 73). For example, Dosenbach *et al*. (72) suggested that these networks communicate with each other, and that each of them carries out dissociable control functions, such as adaptive control in the FPN and stable set-maintenance functions in the CON. Furthermore, the interaction between FPN and CON may be involved in the integration of salient cognitive and affective information to promote goal-directed behavior (74, 75). Thus, we cautiously speculate that the tendency toward higher social comparison can be characterized in terms of increased FC between the CON and FPN, and that this increased FC integrates affective and cognitive/comparative information in the pursuit of self-promotional goals, even during resting periods. Given that the SCO showed negative association with VS–MPFC FC and positive association with IPS–aINS/amygdala FC, we also speculate that individuals with higher social comparison operate more within the external valuation system (IPS-aINS/amygdala FC) and less within the internal valuation system (VS–MPFC FC) than those with lower social comparison during rest. However, there is a lack of additional data supporting this speculation, so future studies should clarify this issue by using functional neuroimaging data obtained simultaneously with behavioral indices of social comparison.

For the exploratory whole-brain analyses, significant relationships of SCO were found only with the RS-fMRI measures but not with the DTI measures. While DTI measures quantify properties related to the direct anatomical links (i.e., white matter fibers) between voxels, RS-fMRI measures quantify the voxel itself and local or remote connections between voxels, especially in the absence as well as in the presence of direct anatomical links. From this point of view, our results for IPS connectivity may reflect FC derived from indirect anatomical connections (76). ROI approach has the advantage of alleviating the multiple comparisons problem by the limiting the number of statistical tests when there are specific hypotheses. Therefore, because of the aforementioned advantage, it may be that the relationship with SC in the present study was found in ROI analysis, but not in voxel-level analysis. In this regard, another possible interpretation is that our DTI measures may be less sensitive in detecting relationships with SCO at the whole-brain level due to more stringent threshold. SC estimation is challenging owing to complex fiber orientations, such as crossing fibers within a voxel. This problem may cause false-positive and false-negative connections, generating spurious and overlooked links of fiber tracts, respectively. In this regard, the current spatial resolution and analytical techniques for DTI data are not sufficient to solve the issue referred to as the “crossing-fiber problem.” Future studies using data with more gradient directions (e.g., high angular resolution diffusion imaging, HARDI) (77) and multiple tensor models (e.g., Q-ball) (78) will clarify the relationship between SCO and SC without the crossing-fiber problem.

The present study had some limitations that should be addressed in future research. Firstly, our interpretations of the findings were necessarily limited by the paucity of information about the directionality of SC and FC. Secondly, it is unclear whether the observed associations reflect the causes or the results of the different levels of social comparison, mainly because the study was cross-sectional in design. The strength of VS–MPFC FC during rest declines with age (79), so future research with longitudinal design should address whether the observed associations change with age. Finally, because the exploratory nature of additional analyses to test whether SCO is associated with certain areas and networks outside the reward networks, hence no further correction for the number of all analyses performed (including mediation analysis, SCO and inter-network connectivity, and regression models) was performed, though each of all these separate analyses was corrected for multiple comparisons.

In conclusion, to our knowledge, the present study was the first to demonstrate that task-independent neural markers can explain individual variabilities in social comparison. Using multimodal, task-independent neuroimaging data, including DTI and RS-fMRI data, we identified several brain networks associated with individual differences in SCO, including the reward network (comprising the MPFC and VS), the FPN (containing the IPS), and the CON (containing the aINS/amygdala). These networks have previously been implicated in either social comparison or general comparative information processing. The present study provides novel and important insights regarding the neural mechanisms underlying individual differences in SCO, suggesting that social comparison is a multidimensional process that engages the networks associated with various motivational, affective, and cognitive components.

## Data Availability Statement

All datasets presented in this study are included in the article/**Supplementary Material**.

## Ethics Statement

The studies involving human participants were reviewed and approved by Korea University. The patients/participants provided their written informed consent to participate in this study.

## Author Contributions

HK and WJ designed the research. WJ analyzed the data. All authors contributed to the article and approved the submitted version.

## Funding

This work was supported by a grant from the National Research Foundation of Korea, which is funded by the Korean Government (NRF-2017M3C7A1041822, to HK and WJ), as well as by a Research Grant from Korea University (to WJ).

## Conflict of Interest

The authors declare that the research was conducted in the absence of any commercial or financial relationships that could be construed as a potential conflict of interest.
