# Supplementary figures and images for: Intrinsic Functional and Structural Brain Connectivity in Humans Predicts Individual Social Comparison Orientation
Source: Front Psychiatry. 2020 Aug 13;11:809. doi: 10.3389/fpsyt.2020.00809 (PMC7438712; doi:10.3389/fpsyt.2020.00809)

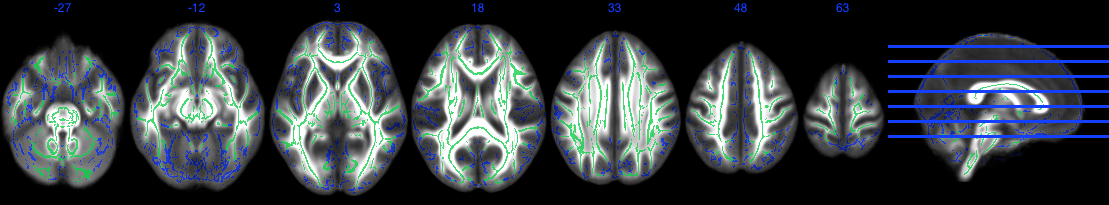

Supplement: Supplementary file 1 [file Image_1.png]
